# High Resolution *In Vivo* Bioluminescent Imaging for the Study of Bacterial Tumour Targeting

**DOI:** 10.1371/journal.pone.0030940

**Published:** 2012-01-25

**Authors:** Michelle Cronin, Ali R. Akin, Sara A. Collins, Jeff Meganck, Jae-Beom Kim, Chwanrow K. Baban, Susan A. Joyce, Gooitzen M. van Dam, Ning Zhang, Douwe van Sinderen, Gerald C. O'Sullivan, Noriyuki Kasahara, Cormac G. Gahan, Kevin P. Francis, Mark Tangney

**Affiliations:** 1 Cork Cancer Research Centre, Mercy University Hospital and Leslie C. Quick Jr. Laboratory, University College Cork, Cork, Ireland; 2 Caliper – a PerkinElmer Company, Alameda, California, United States of America; 3 School of Medicine, University of California Los Angeles, Los Angeles, California, United State of America; 4 Department of Microbiology and Alimentary Pharmabiotic Centre, University College Cork, Cork, Ireland; 5 Department of Surgery, Division of Surgical Oncology, BioOptical Imaging Center, University of Groningen, Groningen, The Netherlands; 6 School of Pharmacy, University College Cork, Cork, Ireland; Case Western Reserve University, United States of America

## Abstract

The ability to track microbes in real time *in vivo* is of enormous value for preclinical investigations in infectious disease or gene therapy research. Bacteria present an attractive class of vector for cancer therapy, possessing a natural ability to grow preferentially within tumours following systemic administration. Bioluminescent Imaging (BLI) represents a powerful tool for use with bacteria engineered to express reporter genes such as *lux*. BLI is traditionally used as a 2D modality resulting in images that are limited in their ability to anatomically locate cell populations. Use of 3D diffuse optical tomography can localize the signals but still need to be combined with an anatomical imaging modality like micro-Computed Tomography (μCT) for interpretation.

In this study, the non-pathogenic commensal bacteria *E.coli* K-12 MG1655 and *Bifidobacterium breve* UCC2003, or *Salmonella* Typhimurium SL7207 each expressing the *luxABCDE* operon were intravenously (IV) administered to mice bearing subcutaneous (s.c) *FLuc*-expressing xenograft tumours. Bacterial *lux* signal was detected specifically in tumours of mice post IV-administration and bioluminescence correlated with the numbers of bacteria recovered from tissue. Through whole body imaging for both *lux* and *FLuc*, bacteria and tumour cells were co-localised. 3D BLI and μCT image analysis revealed a pattern of multiple clusters of bacteria within tumours. Investigation of spatial resolution of 3D optical imaging was supported by *ex vivo* histological analyses. *In vivo* imaging of orally-administered commensal bacteria in the gastrointestinal tract (GIT) was also achieved using 3D BLI. This study demonstrates for the first time the potential to simultaneously image multiple BLI reporter genes three dimensionally *in vivo* using approaches that provide unique information on spatial locations.

## Introduction

The study of bacteria in small animal models is of high importance to a range of medical research fields, including infectious diseases, gut health and gene therapy. The value of non-invasive, longitudinal monitoring of bacterial strains *in vivo* is well accepted, not only because such methodologies drastically reduce animal usage, but also the data generated are more statistically relevant than end-point assays. The bacterial cells, kinetics of transgene expression and host cell responses may be quantitatively assessed simultaneously over time. BLI is based on the detection of bioluminescent light from the subject through use of a cooled charged coupled device (CCD) camera. The relatively simple instrumentation and lack of requirement for radioactivity puts the technology well within the reach of the average laboratory.

Visualisation of bacteria in animal models has become widespread in recent years [1], [2]. The *luxCDABE* operon isolated from *Vibrio harveyi* [3] and *Photorhabdus luminescens* (formerly *Xenorhabdus luminescens*), amongst others, has enabled microbiologists to generate constitutively bioluminescent cells in a diverse range of bacterial genera, without the necessity of exogenous substrate [4]. The first experiments demonstrating *in vivo* detection of a bioluminescent signal were reported in 1995 by Contag and co-workers and involved the imaging of mice infected with *lux*-tagged *Salmonella* Typhimurium [5]. Other luminescent gene systems are also available for bacteria, such as beetle luciferases which have been expressed in *Bifidobacterium longum* [6] and *E. coli* [7]. Strains have also been engineered to express fluorescent markers [8]. However, high background levels and poor tissue penetration of both excitation and emission light restricts the application of fluorescence *in vivo* to predominantly subcutaneous, shallow depth models. Luminescence-based reporter systems display fewer drawbacks, since autoluminescence is almost nonexistent, allowing significantly higher sensitivity and specificity than fluorescent reporters *in vivo*.

In the context of gene therapy, the use of biological agents for delivery of therapeutic genes to patients has shown great promise. Like viruses, the innate biological properties of bacteria permit efficient DNA delivery to cells or tissues, particularly in the context of cancer. It has been shown that bacteria are naturally capable of homing to tumours when systemically administered resulting in high levels of replication locally, either external to (non-invasive species) or within tumour cells (pathogens). This was originally established following IV administration of species of *Clostridium*, and in more recent years with numerous bacterial species, including *Salmonella*, *Bifidobacterium*, *Escherichia coli*, *Vibrio cholerae* and *Listeria monocytogenes* [9], [10], [11], [12]. Strains of clostridia [13] and salmonella have been investigated in clinical trials involving tumour targeting [14], [15], [16]. Various factors are believed to be involved in the tumour-specific nature of bacterial growth *in vivo*, including low oxygen potential (hypoxia), irregular blood supply, local immune suppression and unique nutrient availability within necrotic tumour regions [17], [18]. However, the precise mechanisms involved in bacterial tumour targeting are poorly understood and require rigorous research. Live imaging of mice has shown systemically administered *lux*-tagged bacteria replicating locally in tumours [1], [19], [20]. Yu *et al.* first reported *in vivo* BLI of bacteria in tumours growing in mice following intravenous (IV) administration of *lux*-labelled attenuated *S.* Typhimurium and *Vibrio cholerae* [1].

BLI displays many benefits when compared with other *in vivo* modalities. It is easy to use, inexpensive, rapid and facilitates imaging of multiple animals simultaneously, producing little background with high sensitivity. Traditionally, optical images are two-dimensional and thus devoid of depth information. A major limitation relates to poor spatial resolution, light scattering and absorption by the tissue can substantially impact the 2D measurement [21]. A recent comparative study of imaging technologies reported that the BLI signal measured *in vivo* on shaved mice was reduced 3-fold compared with the signal from the same tumour *ex vivo* after excision [22]. Therefore, in our case, a lux signal from deep tissue or within a tumour is dispersed and does not pinpoint the precise source of the signal in a 2D image.

We propose to address these failings through use of 3D optical tomography, in combination with μCT. For these investigations, we employ non-pathogenic commensal bacteria that colonise both the GIT and s.c tumours to high levels. We have engineered a number of strains of *Bifidobacterium* and *E. coli* to express the luxABCDE cassette. *E. coli* is part of the flora of the human GIT. Several studies have outlined the safety of IV administration of non-pathogenic *E. coli* strains to mice, and their ability to grow specifically within tumours [18], [23]. *E. coli* MG1655 (as used in this study) colonises the mouse GIT to high levels [7]. Bifidobacteria are a native, harmless resident of the human gut, and certain bifidobacterial strains have been shown to have health-promoting or probiotic benefits [24]. We have previously shown that *B. breve* UCC2003 colonises mouse GIT [25] and various tumour models in mice, not only post IV administration, but also following oral GIT colonisation of mice [19]. This study examines the intra-tissue growth of these *lux*-labelled bacteria in live mice both two and three-dimensionally using *in vivo* BLI and μCT co-registration.

## Results

### 1. Tumour-Related Growth of Pathogenic and Non-Pathogenic Bacteria Following IV Administration

**Figure 1:**
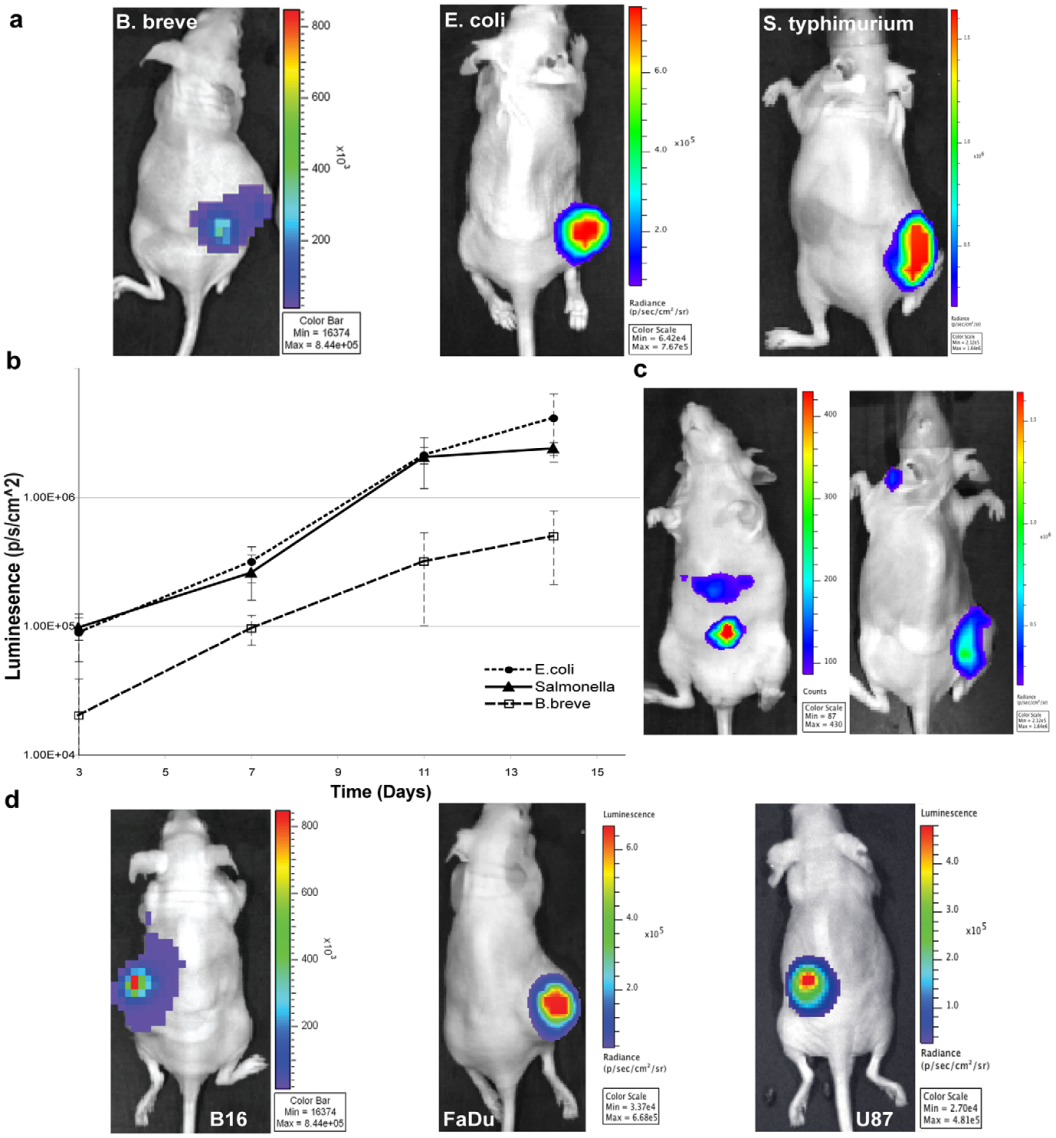
Bacterial Growth in Tumours. (**a,b**) Bioluminescence from *B. breve* UCC2003, *E. coli* MG1655 or *S.* Typhimurium SL7207 in s.c. LLC tumour bearing mice 11 days following IV delivery. Representative mice are shown. Increase in bacterial bioluminescence in tumours was observed over time (n = 6). Data graphed represent the mean ± S.E. There was no detectable bioluminescence in organs of treated animals, except for *S.* Typhimurium (**c**) Representative *S.* Typhimurium administered mice displaying non-tumour-specific bacterial bioluminescence. Ventral image – Day 3, Dorsal – Day 11. (**d**) All tumour types examined were colonised by the various strains. (i) *B. breve*, B16, Day 11 (ii) *E. coli*, FaDu, Day 7 (iii) *B. breve*, U87, Day 14.

Bacterial homing to and replication within various subcutaneous tumours following IV administration was imaged over time using 2D whole body BLI of mice. We compared the tumour-specific replication ability of the non-pathogenic commensal bacteria *E. coli* MG1655 and *B. breve* UCC2003 with that of the most investigated species in the field, *Salmonella* Typhimurium [26], [27]. 1×10^6^ lux-expressing *E.coli* MG1655 or *S.* typhimurium SL7207 or 1×10^5^ *B. breve* UCC2003 (corresponding to the maximum tail-vein injectable dose, in terms of viscosity of bacterial solution) were administered via tail vein injection to athymic mice bearing subcutaneous Lewis Lung Carcinoma (LLC) tumours (n = 6). 2D *in vivo* BLI was performed at various times post bacterial administration. Lux signal was detected specifically in tumours of mice 3–14 days post IV-administration of *B. breve* or *E. coli* (Figure 1a,b). Unlike the non-pathogenic bacteria examined, *S.* Typhimurium-related bioluminescence was also observed in areas other than tumours, in particular, from the abdomen (at day 3) and neck (at day 11) of the infected animals (Figure 1c). This observation correlates well with previous studies, where growth within the liver and spleen [5] was reported.

**Figure 2:**
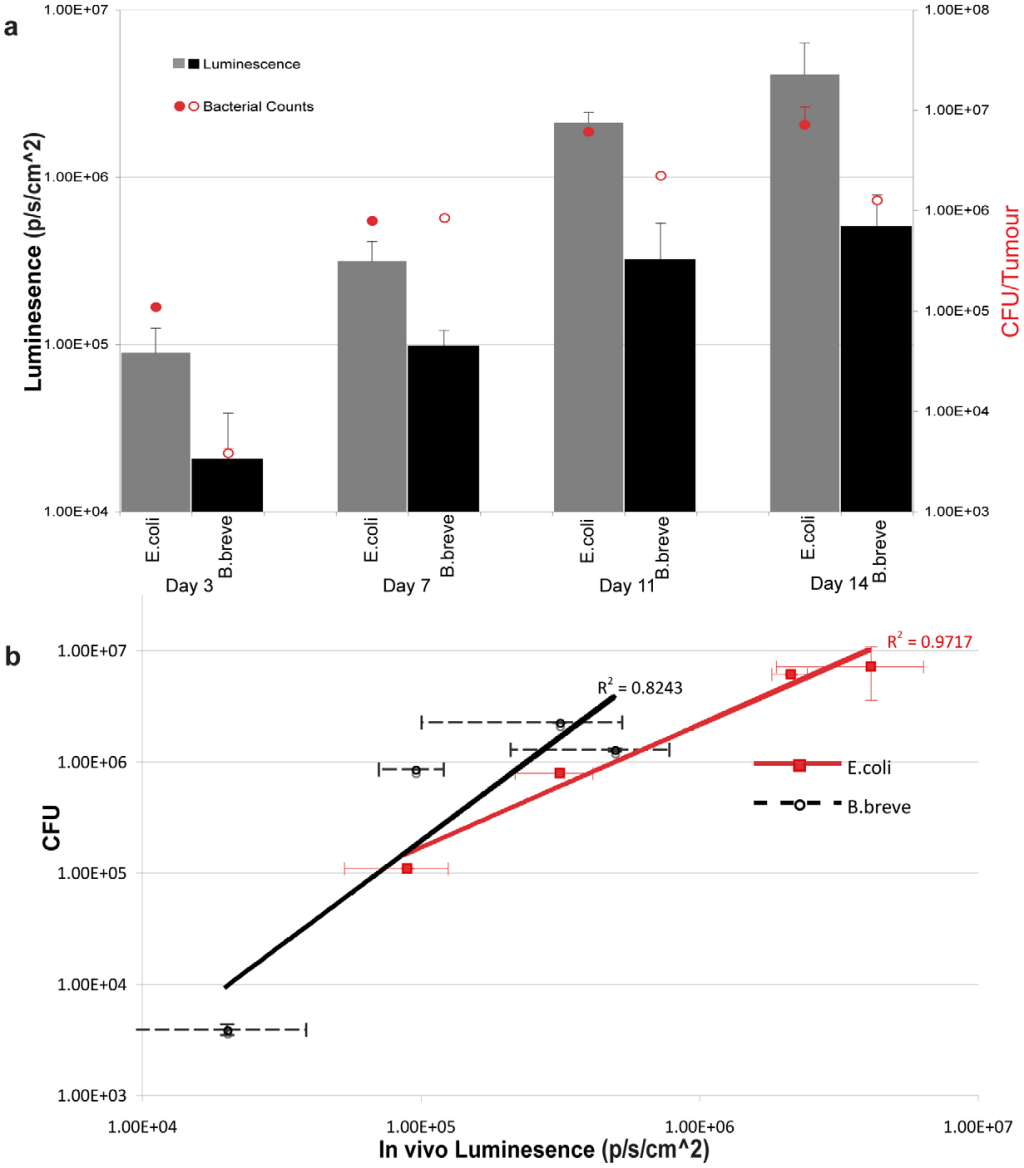
Relationship Between Bacterial Numbers And Bioluminescence. Viable bacteria in tumours were enumerated by *ex vivo* bacterial culture from LLC tumours subsequent to BLI at various time-points post IV administration of *B. breve* or *E. coli* (n = 6). (**a**) **Bacterial replication in tumours.** Increases over time in viable bacterial numbers and bioluminescence. Data graphed represent the mean ± S.E. (**b**) **Correlation between bacterial numbers and bioluminescence in tumours.** Direct comparison between photon flux and bacterial colony counts. Log values of bacterial numbers relative to *in vivo* bioluminescent units are graphed. Correlation between bacterial counts and bacterial bioluminescence signals: R^2^ = 0.84 for *B. breve*: R^2^ = 0.97 for *E. coli* which correlates well with previous studies using *E. coli* MG1655 where R^2^ = 0.94, *P*<0.001 [57].

Subsequent BLI studies proceeded with the non-pathogenic commensal bacteria. Bacterial replication in tumours was confirmed by *ex vivo* bacterial culture and viable bacterial numbers correlated with bioluminescence for each strain (Figure 2). Each strain displayed characteristic levels of bioluminescence in tumours (*B. breve* = 0.128±0.014; *E. coli* = 0.373±0.025 flux units per cfu, on days 7–11). The relationship between bioluminescence and bacterial counts followed linear trends (R^2^ values shown in Figure 2). Because the activity of lux bioluminescence is dependent on both FMNH_2_ and oxygen availability, lower bioluminescence/cfu may be the result of decreased metabolic activity and/or reduced oxygen availability at higher bacterial concentrations and/or larger more hypoxic tumours.

To examine the range of tumour types applicable to bacterial targeting, MG1655 or UCC2003 were IV administered to mice bearing s.c. murine B16 melanoma, human U87 glioblastoma or human FaDu hypopharyngeal carcinoma xenograft tumours. Tumours were colonised in all cases, indicating that tumour colonisation is independent of tumour type (Figure 1d).

### 2 Two- and Three-dimensional Bioluminescent Imaging of Non-pathogenic Bacteria Following Oral Administration

**Figure 3:**
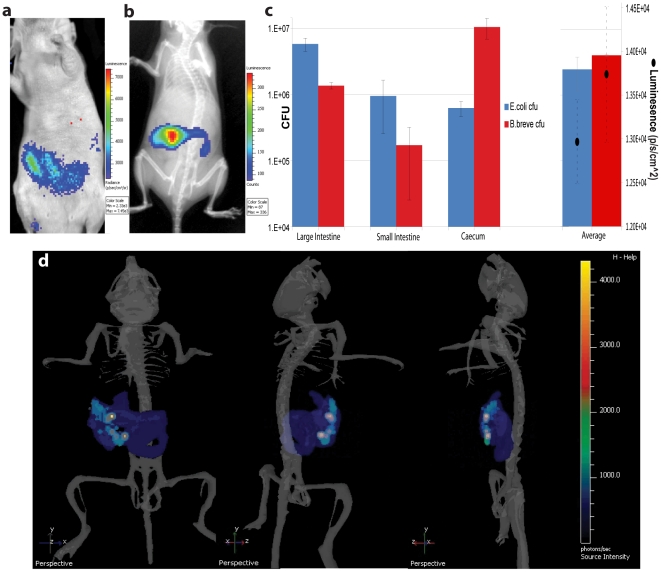
Bacterial Bioluminescence in the GIT. The GIT of mice was colonised with either *B. breve* or *E. coli* by oral administration of 10^9^ cfu of the relevant strain for three consecutive days. (**a**) 2D image of athymic mouse 9 days post final feed with *B. breve* UCC2003/lux. (**b**) IVIS XR 2D bioluminescence overlayed with X-ray image of mouse 27 days-post feeding with *E. coli* MG1655/lux. (**c**) Bacterial counts in specific regions of the GIT 14 days post feeding. Abdominal bioluminescence corresponding to average cfu is also shown (black dots). Data graphed represent the mean ± S.E. (**d**) Sample isolated images from 3D tomography of mouse from (b). 3D images show a digital mouse atlas of the skeleton to provide anatomical registration. (Movie available in Movie S1). *E. coli* MG1655 bioluminescence is visible in blue at lower, and white or green at higher levels.

We have previously shown that oral administration of *B. breve* UCC2003 leads to s.c. tumour colonisation at levels similar to IV administration [19]. We routinely utilised bacterial culture to demonstrate high-level GIT colonisation following oral gavage with this strain [25]. Here, we examined the ability to *in vivo* image bacteria within the GIT. The 2D image in Figure 3a indicates the presence of *B. breve* UCC2003 in the abdominal region 9 days post oral administration. Similarly, *E. coli* MG1655 was readily 2D imaged in the region of the GIT post oral administration (Figure 3b), with a more defined/localised pattern of light due to the higher bioluminescence emitted by this strain. Diffuse optical tomography (Figure 3d, Movie S1) in combination with a GIT segmented from the mouse anatomical atlas revealed *E. coli* luminescence localised in the general area of the GIT. Peak luminescence levels were observed within the large intestine region, correlating with results obtained by sampling and culturing bacteria from different regions of the GIT (Figure 3c). A movie depicting rotational 3D tomography can be seen in (Movie S1).

### 3 Co-localisation of Bacterial lux and Tumour-related FLuc Bioluminescence

**Figure 4:**
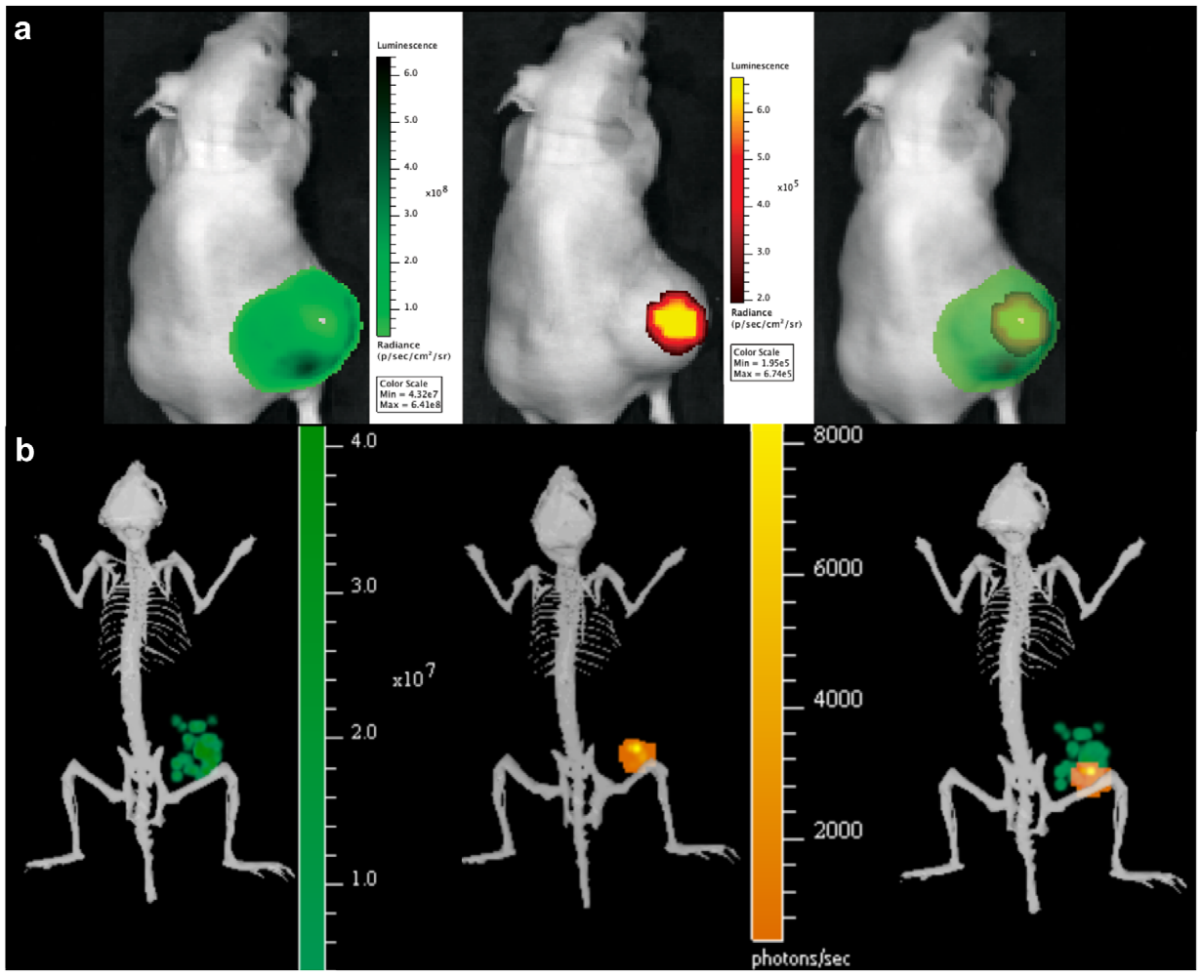
Co-localisation of Tumour And Bacterial Bioluminescence. (**a**) **2D bioluminescence co-registration.** Athymic mouse bearing s.c. FLuc expressing FaDu tumour 7 days post IV administration of *B. breve*. Tumour FLuc = Green, Bacterial lux = orange. (**b**) **3D bioluminescence co-registration.** 3D overlay of *B. breve* lux (orange) 10 days post administration to athymic mice bearing HCT116 FLuc (green) expressing tumours.

**Figure 5:**
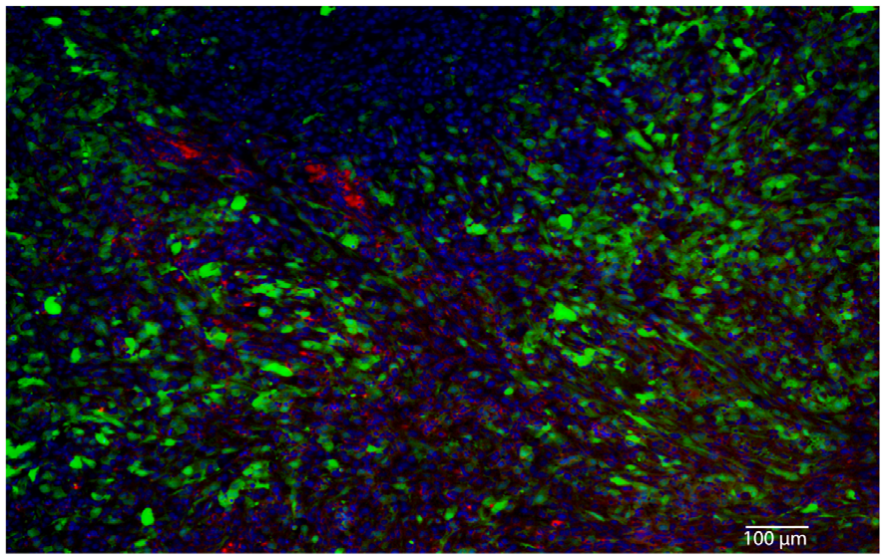
*Ex vivo* histological analysis. Fluorescent bacterial and tumour cells were imaged using fluorescence microscopy (Nuance). Blue- DAPI (nuclei), Green- U87/GFP, Magenta- *B. breve*/pCheMC.

For cancer gene therapy, the ability to establish vector targeting of malignant versus healthy tissue is of immense value. Through sequential whole body imaging for tumour-associated FLuc and bacterial lux signals, we demonstrate the potential for co-localising these bioluminescent signals. Figure 4a shows a representative 2D image overlay of tumour and bacterial bioluminescence, demonstrating the location of each specifically in the tumour region. 2D imaging lacks depth information, and therefore the pattern of surface bioluminescence in the image output appears to be dispersed throughout the tumour. Figure 4b shows sample 3D images, providing tomographic detail of source signal distribution. 3D information indicates non-uniform distribution of both bacterial and tumour signals. *B. breve* lux was observed in multiple disparate ‘clusters’ within the tumour (Movie S2). *Ex vivo* imaging of tissue sections of cherry fluorescent protein-expressing *B. breve*/pCheMC and GFP-expressing tumours (U87-fLuc2/GFP) (Figure 5), confirmed the non-uniform distribution of *B. breve*, primarily in less viable regions of tumour, as evidenced by low levels of GFP signal surrounding the bacterial cherry signal, in tissue sections taken from the centre of tumours (Figure 5). By contrast, no cherry signal was visible in tissue sections taken at the periphery of tumours, which were highly populated with GFP-positive cells (data not shown). Immunohistochemical staining for endothelial cells (CD31) further highlighted the concentration of vasculature in regions of higher viable tumour cell density (Figure S1).

### 4 Co-registration of BLI and μCT

**Figure 6:**
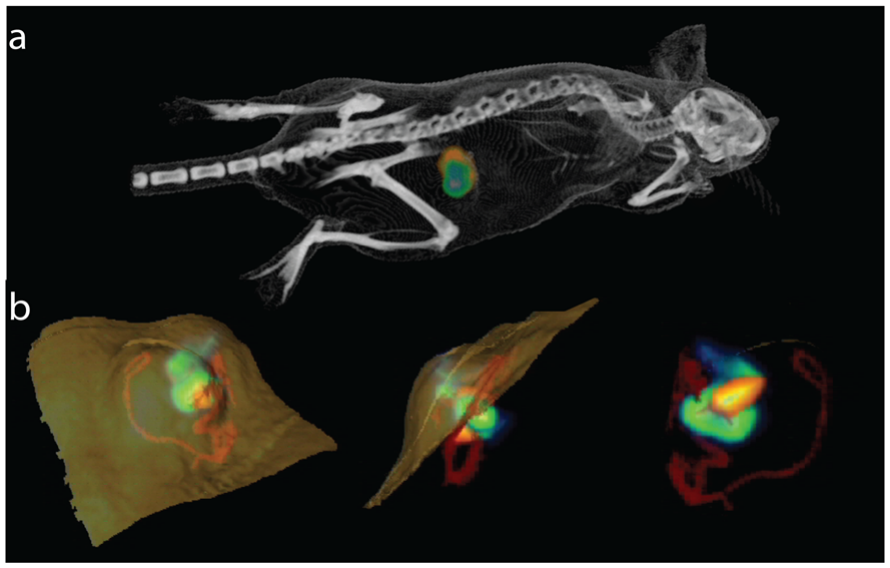
Co-registration with μCT. (**a**) **Whole Body 3D co-registration of lux, FLuc and μCT.** Combined luminescence and μCT demonstrating co-localisation of *B. breve* (bacterial lux - orange) and subcutaneous HCT116-luc2 tumour (FLuc - green). (**b**) **Intratumoural Imaging.** Combined μCT and luminescence imaging. Magnification of subcutaneous tumour from mouse in (a), top, side and base view. Viable tumour (FLuc green/blue), vasculature (contrast agent – red) and bacterial (orange/yellow) signals are visualised. Movie on website (Movie S3).

Mice bearing s.c. HCT116-luc2 tumours (average volume 120 mm^3^) 10 days post administration with *B. breve* UCC2003 were 3D BLI imaged, followed immediately by μCT imaging. Co-registration of 3D BLI and μCT facilitated the anatomical positioning of bioluminescent signal sources within the tumour (Figure 6), in addition to visualisation of tumour vasculature through administration of a contrast agent. Software image analysis focussed on the tumour permitted partial assessment of the intra-tumoural location of bacteria (Figure 6b, Movie S3). A portion of the bacterial signal appeared to be co-localized within the tumour with a rendering of the main tumour vasculature around the tumour base.

## Discussion

While determination of bacterial distribution and cell numbers present over time can be achieved to some extent through the use of traditional *ex vivo* analytical methods (culture, histology etc.), these end-point assays are labour-intensive and expensive, requiring the death of large numbers of animals, with high-throughput experimentation difficult to achieve [28]. We demonstrate here, the usefulness of BLI in permitting *in vivo* detection of viable bacteria, empowering the investigator with the ability to non-invasively assess bacterial trafficking and growth over time with precision. We assessed the trafficking of two non-pathogenic strains (a Gram-positive and a Gram-negative genus) in tumour-bearing mice, and compared these data with those obtained using the ‘gold-standard’ cancer therapy strain *S.* Typhimurium.

Various preclinical therapeutic trials have shown the ability of different bacterial strains to traffic to tumour sites, primarily in the context of delivery of DNA for subsequent tumour cell expression. Pathogenic invasive species are utilised for this purpose [29]. *S.* Typhimurium has been examined in clinical trials for tumour targeting [14], [15], [16]. However, even with safety attenuation, the inherent pathogenicity and immunogenicity of these bacteria has outweighed therapeutic responses in patients. To address these failings, we explored the use of non-pathogenic bacteria and validated that these strains colonise tumours with efficiencies similar to the best-described species in the field, *S.* Typhimurium. The *E. coli* and *B. breve* strains employed here are non-invasive. In the context of gene and cell therapy, such bacteria do not act as cell transfection agents, but rather replicate within the tumour stroma, external to tumour cells. Therapeutic strategies employing these bacteria are analogous to cell therapy approaches, with bacterial expression of therapeutic genes (e.g. anti-angiogenic or immune modulating) within the environment of the tumour [10]. The non-invasive nature of these strains bestows further tumour-specificity and safety, as was indicated here with *B. breve* and *E. coli*, where their bioluminescence was confined to just tumours, unlike that of *S.* Typhimurium which was also observed elsewhere in the animal.

The *E. coli* and *S.* Typhimurium strains in this study were labelled by genomic integration of the *lux* operon. p16sLux integrates in single copy into the 16s rDNA site in the chromosome of Gram-negative strains [30]. The use of a site-specific integrating plasmid permits labelling of bacteria without interference in the behaviour of the strain following integration. Bifidobacteria are much less genetically tractable, and such genomic integration strategies are unavailable. The lower levels of bioluminescence observed from *B. breve* are likely to reflect lower lux gene expression compared with *E. coli* and *Salmonella*, given the nature of the episomal plasmid construct, but might also be reflective of lower metabolic activity within this particular bacterium (this is currently being investigated). Nonetheless, the pMC3 plasmid-based *lux* system used here proved sufficiently robust to permit BLI tracking in a range of situations, and this system is still the only reported *luxABCDE* expression system for bifidobacteria [25]. Fluorescent protein tagging also proved useful in this study for *ex vivo* analysis of *B. breve* in tumour tissue, since to date we have been unable to visualise this strain in tumours by Gram-staining. Use of the cherry red fluorescent strain, in conjunction with a green fluorescent tumour cell line validated and provided further detail on *in vivo* findings with 3D BLI.

While other studies using 2D BLI have demonstrated localisation of bacteria to various regions of the mouse body (abdomen, lung, s.c. tumour etc.), no depth information is available with 2D imaging. The 2D image obtained is at the level of the animal surface and is dispersed over an area broader than the source. This poor spatial resolution limits conclusions that can be drawn. This is exemplified in Figure 4a, where 2D imaging suggests that bacteria are distributed throughout the tumour, whereas 3D tomography (Movie S2) elucidates brighter source locations and implies that the bacteria are concentrated non-uniformly within the tumour mass. The histological analysis confirmed the presence of *B. breve* in clusters, primarily in less viable regions of tumour as evidenced by less green cells. While DAPI-stained nuclei were present proximal to bacteria, the absence of GFP suggests that DAPI-positive cells are not metabolically active U87 cells, and may relate to other cell types of tumour stroma, such as immune or fibroblast. It should be noted that tissue sections were 6 µm in thickness, and therefore not all cells visualised are in the same plane.

Bifidobacterial growth in non-viable tumour regions has previously been established by us and others. As tumours grow over 2 mm^3^, the vascular supply becomes inadequate to supply metabolic demands, resulting in areas of hypoxia. There are multiple areas in tumours with <1% oxygen compared with a standard 3–15% in normal tissues. This is apparent with 3D imaging in Figure 4b, where FLuc bioluminescence from U87 tumours is seen to be non-uniform throughout the tumour. Early observations with strictly anaerobic bacteria (clostridia and bifidobacteria) lead to the hypothesis that, unlike normal tissues, hypoxic environments in tumours provide anaerobic growth conditions resulting in tumour-specific bacterial growth [31], [32]. However, the precise mechanism of tumour-specific bacterial growth has yet to be demonstrated. Entry, survival and replication of bacteria in tumours may also involve the leaky vasculature of tumours and the tumour immune microenvironment which provides a sanctuary for bacteria to escape the immune system [1]. The nutrient rich environment created by cell death in necrotic regions may also play an important role as evidenced by findings that tumours can support the replication of auxotrophic strains of *S.* Typhimurium like the SL7207 examined here [27], [33], [34]. It has been reported for many species, including strains of *S.* Typhimurium, that bacterial growth was confined to necrotic regions of tumours, while in contrast, certain strains, such as *S.* Typhimurium A1, have been shown to grow throughout the tumour, including viable malignant tissue in a range of tumour models [27], [33], [34], [35], [36], [37]. In our previous studies, UCC2003 was detected solely in necrotic regions, as evidenced by PCR [19]. 3D BLI combined with CT imaging provides *in vivo* evidence of this here, with *B. breve* lux concentrated within the tumour. While there was not enough resolution to detect capillaries and microvasculature, larger vessels (near the tumour base) could be readily resolved and segmented for visualization by μCT.

This study is not the first attempt to register 3D optical and μCT images. Some studies have looked at specific integrated instrumentation to combine fluorescence and μCT [38], [39]. Other studies have taken approaches similar to ours and transferred animals between separate instruments and performed the registration afterwards [40], [41]. As with any unique imaging approach there are limitations. The diffuse optical tomography reconstruction algorithm used here assumes that tissue optical properties are homogenous [21]. For the data presented here, GIT imaging (Figure 3d) may also be biased by registration errors on the order of 0.5 mm by going between two instruments [41]. When comparing optical reconstructions to the anatomical atlas, visual interpretation at a gross scale provides reference of a bioluminescent source reconstruction to the general anatomy. For the subcutaneous 3D optical data (Figure 6), the spatial positioning of the 3D optical signals has been shown to be accurate to the order of 1–2 mm [24], [42].

Though oxygen is involved in the lux enzymatic reaction, a number of anaerobic bacteria have been successfully labelled with *lux*, and shown to produce bioluminescence under the normal low oxygen/anoxic growth condition of these bacteria [43]. Moreover, bioluminescence from a number of pathogenic bacteria in the supposed anaerobic environment of the GIT has been detected by 2D BLI [44], [45], [46]. In this study, we validate the functioning of lux at low to negligible oxygen tensions. We imaged both commensal strains in the mouse GIT in this study. 2D *in vivo* imaging of another commensal *E. coli* strain, *E. coli* Nissle, has recently been reported [47]. We report here, the novel ability to sub-localise GIT regions of highest colonisation using 3D BLI.

Overall, we demonstrate the potential for non-pathogenic bacteria as vectors for cancer therapy. Analysis of reporter gene activity from these strains not only demonstrates their potential as safe and non-invasive vectors, with the potential to deliver therapeutic or diagnostic agents systemically but furthermore underlines the significance of BLI and μCT co-registration as tools to advance this concept.

## Materials and Methods

### Bacterial Strains

*B. breve* UCC2003 (UCC Culture Collection) was routinely grown at 37°C in reinforced clostridial medium (RCM) (Oxoid, Basingstoke, United Kingdom). For bioluminescence assays and preparation of the bacteria for animal inoculations MRS medium (Oxoid), supplemented with 0.05% (w/v) cysteine-HCl was used. Anaerobic conditions were maintained using an anaerobic chamber (Mac500, Don Whitley Scientific, West Yorkshire, UK (atmosphere 10% H_2_, 10% CO_2_, 80% N_2_). *B. breve* UCC2003/pLuxMC3 [25], expressing the plasmid based *lux*ABCDE operon from the P*_help_* promoter [30] was cultured in the presence of 4 µg/ml chloramphenicol (Cm). To facilitate specific recovery of bifidobacteria from tissue samples, 50 mg mupirocin (Oxoid)/litre was included, as previously described [48].

An mCherry fluorescent *B. breve* UCC2003 was also generated. 700 base pair coding region encompassing the mCherry gene was amplified by PCR from an mCherry plasmid obtained from Roger Tsien [49], digested with KpnI and BamHI and ligated to a similarly digested pSKEm [50], to generate pSKcherry. The putative promoter region of *repC* from the *B. catenulatum* plasmid pBC1 [51] was PCR amplified and digested with XhoI, and ligated to pSKcherry digested with XhoI/EcoRV creating pCheMC, before transformation of UCC2003 by electroporation.

*E. coli* K-12 MG1655 [52] containing the integrated *luxABCDE* was grown aerobically at 37°C in LB medium (Sigma, Steinheim, Germany) supplemented with 300 µg/ml erythromycin (Em). The bioluminescent derivative of MG1655 was created using the plasmid p16S*lux* which contains the constitutive P_HELP_ *luxABCDE* operon [30] on the backbone of pGh9::IS*S1*, a thermo-sensitive shuttle vector which integrates randomly into the bacterial chromosome as a consequence of the presence of IS*S1* [53]. p16S*lux* was transformed into MG1655 by electroporation using standard protocols essentially as described by Riedel *et al.* [54]. Integration of the plasmid was achieved by incubation of isolated positive clones at temperature non-permissive to plasmid replication (42°C) under antibiotic selective pressure (erythromycin, Em). Em^R^ colonies were checked for light emission, and the integration of p16S*lux* was confirmed by PCR using primers 16S_rev_XhoI and 16S_fwd_int [54] yielding the expected 1,150-bp fragment.

The auxotrophic attenuated strain of *Salmonella enterica* serovar Typhimurium designated SL7207 was received as a kind gift from Dr. Bruce Stoker [55]. This strain was also transformed with p16S*lux* by the same method, as previously described by our group [56].

### Tumour Cell lines and Culture

Murine Lewis Lung Carcinoma cells, B16-F10 cells and human U87 glioblastoma cells (ATCC) were maintained in culture at 37°C in a humidified atmosphere of 5% CO2, in Dulbecco's Modified Essential Medium (GIBCO, Invitrogen Corp., Paisley, Scotland) supplemented with 10% iron-supplemented donor calf serum (Sigma Aldrich Ireland, Ireland), 300 µg/ml L-glutamine and 100 U/ml penicillin and 100 µg/ml streptomycin.

FaDu hypopharyngeal carcinoma (ATCC) was maintained in culture at 37°C in a humidified atmosphere of 5% CO2, in Essential Medium Eagles Modification (Sigma) supplemented with 10% iron-supplemented donor calf serum (Sigma). The HCT116-luc2 cell line (Caliper) was maintained in McCoy's 5a Medium Modified (ATCC) supplemented with 10% foetal calf serum (FCS), 100 U/ml penicillin, 100 µg/ml streptomycin, 2 mM L-glutamine and sodium pyruvate and grown at 37°C in a humidified atmosphere of 5% CO_2_.

U87 cells stably expressing fLuc2 and GFP (U87-fLuc2/GFP) were generated by overnight infection using an HIV-1-based bicistronic lentiviral vector encoding both the firefly luciferase and GFP genes under the control of the CMV promoter, with the GFP gene translated from an internal ribosomal entry sequence (IRES). Greater than 90% transduction efficiency was confirmed by FACS analysis for GFP expression.

### Ethics Statement

All murine experiments were approved by the animal ethics committee of University College Cork or Caliper IACUC animal ethics committee. (AERR #2010/003 and “The use of bacteria as gene vectors in anticancer therapy”.)

### Animals and Tumour Induction

Mice were kept at a constant room temperature (22°C) with a natural day/night light cycle in a conventional animal colony. Standard laboratory food and water were provided *ad libitum*. Before experiments, the mice were afforded an adaptation period of at least 7 days. Female mice in good condition, without fungal or other infections, weighing 16–22 g and of 6–8 weeks of age, were included in experiments. For routine tumour induction, the minimum tumourigenic dose of cells suspended in 200 µl of serum-free culture medium was injected subcutaneously (s.c.) into the flank of 6–8 week old female C57 or athymic MF1-nu/nu mice (Harlan, Oxfordshire, UK) (1×10^6^ B16-F10 tumour cells, 5×10^5^ LLC) or athymic Crl∶NU(NCr)-Fox1nu mice (Charles River) (5×10^5^ U87, 7.5×10^6^ HCT116). The viability of cells used for inoculation was greater than 95% as determined by visual count using a haemocytometer and Trypan Blue Dye Exclusion (Gibco), or the Nucleocounter system (ChemoMetec, Bioimages Ltd, Cavan, Ireland). Following tumour establishment, tumours were allowed to grow and develop and were monitored twice weekly. Tumour volume was calculated according to the formula *V* = (*ab* ^2^) Π/6, where *a* is the longest diameter of the tumour and *b* is the longest diameter perpendicular to diameter *a*. When tumours reached approximately 100 mm^3^ in volume, the mice were randomly divided into experimental groups.

### Bacterial Administration

Inocula were prepared by growing *B. breve* UCC2003/pLuxMC3 anaerobically overnight at 37°C in 100 ml of MRS broth containing 4 µg/ml Cm and *E. coli* MG1655-*luxABCDE* and SL7207 aerobically in 100 ml LB broth containing 300 µg/ml Em. Cultures were harvested by centrifugation (6,000×*g* for 5 min), washed three times with PBS (PBS supplemented with 0.05% cysteine HCl (Sigma) for bifidobacteria), and resuspended in a one-tenth volume of PBS. The viable count of each inoculum was determined by retrospective plating on Reinforced Clostridial Agar (RCA) containing 4 µg/ml Cm for *B. breve* or LB agar containing 300 µg/ml Em for *E. coli* and SL7207.

For GIT colonisation studies, 10^9^ bacterial cells were orally administered in 100 µl per mouse by gavage, on three consecutive days. In the case of *E. coli* studies, pre-existing commensal bacterial levels were decreased prior to feeding by addition of 5 mg/ml streptomycin in mouse drinking water for 7 days prior to commencement of oral gavage.

For tumour-related studies, mice were randomly divided into experimental groups when tumours reached approximately 100 mm^3^ in volume, and administered either *B. breve*, *E. coli*, SL7207 or an equal volume of PBS as control. Each animal received either 10^5^ *B. breve* or 10^6^ *E. coli* or 10^6^ SL7207 in 100 µl injected directly into the lateral tail vein.

### Bacterial Recovery From Mice

At defined time points (Figure 1) following whole-body imaging, a subset of animals from each group were euthanised by cervical dislocation. Individual tumours as well as lungs, liver, spleen and kidneys were aseptically removed. Each tissue was homogenized by fine mincing by scalpel followed by pushing through a 20 µm pore nylon filter (Falcon, Becton Dickinson (BD), Oxford, England) in sterile PBS (supplemented with 0.05% cysteine-HCl for bifidobacteria). Serial dilutions were plated in triplicate on selective agar; *B. breve* on RCA agar containing 4 µg/ml Cm and mupirocin and *E. coli* on LB agar containing 300 µg/ml Em. Resulting colonies were used to calculate the number of bacterial cells per tissue sample. To confirm that cfu recovered were either *B. breve* containing pLuxMC3 or *E. coli* MG1655*luxABCDE*, random isolates were spot inoculated onto the appropriate agar either with or without the selective antibiotic.

### Optical Image Acquisition And Image Formation

2D *in vivo* BLI imaging was performed using the IVIS100 (Caliper). At defined time points post bacterial administration, animals were anesthetised by intraperitoneal administration of 200 mg xylazine and 2 mg ketamine, and whole-body image analysis was performed in the IVIS 100 system for 2–5 minutes at high sensitivity. Regions of interest were identified and quantified using Living Image software (Caliper).

For 3D imaging, mice were anesthetized using Caliper's XGI-8 Gas Anesthesia System with 3% Isofluorane and placed in a mouse imaging shuttle inside of the optical imaging system for dorsal imaging (IVIS Spectrum, Caliper). To acquire images of the bacterial luciferase signal for 3D optical reconstruction, emission filter wavelengths ranging from 500–580 nm were used with bin 16 acquisition times of 3–4 min per filter to maximize the signal to noise ratio. To acquire images of the firefly luciferase emanating from the tumour, luciferin was injected approximately 10 minutes prior to imaging using a subcutaneous approach without moving the animal. Emission filter wavelengths ranging from 580–620 nm were then used with bin 8 acquisition times of 0.5–0.75 min per filter. As part of this image acquisition sequence, a structured light image was obtained to define a height map. This map was input diffuse light imaging tomography (DLIT) reconstructions algorithms that were used to form a 3D optical image using a non-negative least squares optimization [21].

### μCT Image Acquisition and Generation

While mice were still positioned in the shuttle, a preclinical blood pooling contrast agent (exitron nano 12000, Miltenyi, Auburn, CA, USA) was injected via the tail vein. The mouse was then transferred to an *in vivo* μCT imaging system (Quantum FX, Caliper) and the shuttle was positioned in a customized bed containing a fiducial. One image of the entire mouse was acquired and reconstructed into a 12 cm axial FOV image with an isotropic voxel size of 295 um. To obtain a higher resolution image of the tumour for vascular imaging, the mouse was then removed from this shuttle and the magnification was increased to acquiring a 1 cm FOV image of the tumour that was reconstructed with a 20 µm isotropic voxel size. All images were reconstructed using standard back-projection techniques.

### Image Registration and Processing

To co-register the two DLIT 3D optical images with the whole body μCT image, a customized registration routine was implemented to detect the fiducial in the μCT holder for the mouse imaging shuttle (Living Image, Caliper). This algorithm detects the fiducial in the μCT image and then transforms this image to match the identical orientation of the 3D optical image. To overlay these 3D optical images with the high resolution μCT, all images were then imported into a dedicated image processing and visualization package (Avizo Fire, Visualization Sciences Group). Using the whole mouse μCT as a reference frame, the bacterial DLIT, firefly DLIT signal and high-resolution μCT images were separately registered using affine transformations driven by optimization of a normalized mutual information metric. To segment the vasculature in the high resolution μCT image, an edge-preserving smoothing filter was applied prior to use of a threshold limited semi-automatic region growing approach to define the vasculature. A sequence of 3 dilations and 3 erosions was then applied to remove any additional noise. Finally, volume rendering was applied to show this vasculature segmentation and the two DLIT signals overlaid with an isosurface of the skin around the tumour.

### Histological Analysis

For *ex vivo* histological analyses, s.c. U87/GFP tumours were harvested 7, 11 and 14 days post administration with B. breve/pCheMC. Resected tumours were fixed in 10% v/v buffered formalin and embedded in paraffin. Serial 6 µm sections were cut at tumour periphery and tumour centre and mounted on slides. To detect mCherry labelled bacteria and GFP tumour cells on sections, slides were deparaffinised and mounted with DAPI containing media (Vector Lab, Burlingame, CA). GFP, mCherry and DAPI signals were imaged with epifluorescence microscope (Nikon, E400) equipped with a Nuance FX camera (Caliper). Nuance camera contains liquid crystal tuneable filters that can enable spectral unmixing for each fluorophore. Image analysis and spectral unmixing was performed using InForm software (Caliper). Adjacent sections were stained with H&E for morphological analysis.

Vasculature within tumours was examined by immunohistochemistry (IHC) specific for murine CD31 (PCAM-1). Paraffin embedding, sectioning and IHC were carried out by the Translation Pathology Core at UCLA. Tissues were fixed in 10% formalin prior to paraffin embedding. Sections of 4 µm thickness were subjected to immunohistochemical analysis. Briefly, sections were deparaffinized using xylene and rehydrated by passing through ethanol at concentrations ranging from 100% to 50%. Endogenous peroxidase activity was blocked in 3% hydrogen peroxide in methanol for 10 min. Antigen retrieval was achieved by incubation with citrate buffer at 95°C for 25 min. The sections were incubated at RT for 2 hours with PCAM-1 (Santa Cruz Biotechnology, sc-1506) at the dilution of 1∶ 100. The slides were rinsed with PBST (Phosphate Buffered Saline containing 0.05% Tween-20), prior to 30 min incubation at RT with a biotinylated polyclonal Rabbit anti-goat immunoglobulin (Dako, E0466), at a dilution of 1∶200. The sections were rinsed with PBST, and were incubated with Dako EnVision+ System – HRP Labelled Polymer Anti-Rabbit (Dako, K4003) for 30 minutes at RT. The sections were rinsed with PBST then incubated with DAB (3,3′-Diaminobenzidine) for visualisation. The sections were washed in water, counterstained with Harris' Hematoxylin, dehydrated in ethanol, and mounted with media.

### Statistical Analysis

Two-tailed Student's *t*-tests were employed to investigate statistical differences. Microsoft Excel 12 (Microsoft) was used to manage and analyze data. Statistical significance was defined at the standard 5% level.

## Supporting Information

Movie S1 **Bacterial Bioluminescence in the GIT.** Movie depicting rotational 3D optical tomography in combination with mouse anatomical atlas displaying luminescence in GIT of mice 27 days post oral administration of lux-labelled *E. coli* MG1655.
(AVI)

Movie S2 **3D Co-localisation of Tumour And Bacterial Bioluminescence.** Movie providing tomographic detail of source signal distribution of *B. breve* lux (green) 10 days post IV administration to mice bearing HCT116 FLuc (orange) expressing tumours. *B. breve* lux is observed in multiple disparate ‘clusters’ within the tumour.
(MOV)

Movie S3 **3D Intratumoural Optical and μCT Imaging.** Combined μCT and luminescence imaging of subcutaneous HCT116-luc2 tumour colonised by *B. breve*. Viable tumour (FLuc green/blue), vasculature (contrast agent – red) and bacterial (orange/yellow) signals are visualised.
(MOV)

Figure S1 **Tumour Vasculature Immunohistochemistry.** Murine endothelial cells within U87 tumour were visualised by IHC staining specific for CD31. Sections were incubated with anti-PCAM-1 antibody, followed by secondary biotinylated polyclonal Rabbit anti-goat immunoglobulin, and HRP-labelled Polymer Anti-Rabbit and visualised with DAB counterstained with Harris' Hematoxylin. Endothelial cells stain brown.
(TIF)
