# Supplementary figures and images for: High Resolution In Vivo Bioluminescent Imaging for the Study of Bacterial Tumour Targeting
Source: PLoS One. 2012 Jan 25;7(1):e30940. doi: 10.1371/journal.pone.0030940 (PMC3266281; doi:10.1371/journal.pone.0030940)

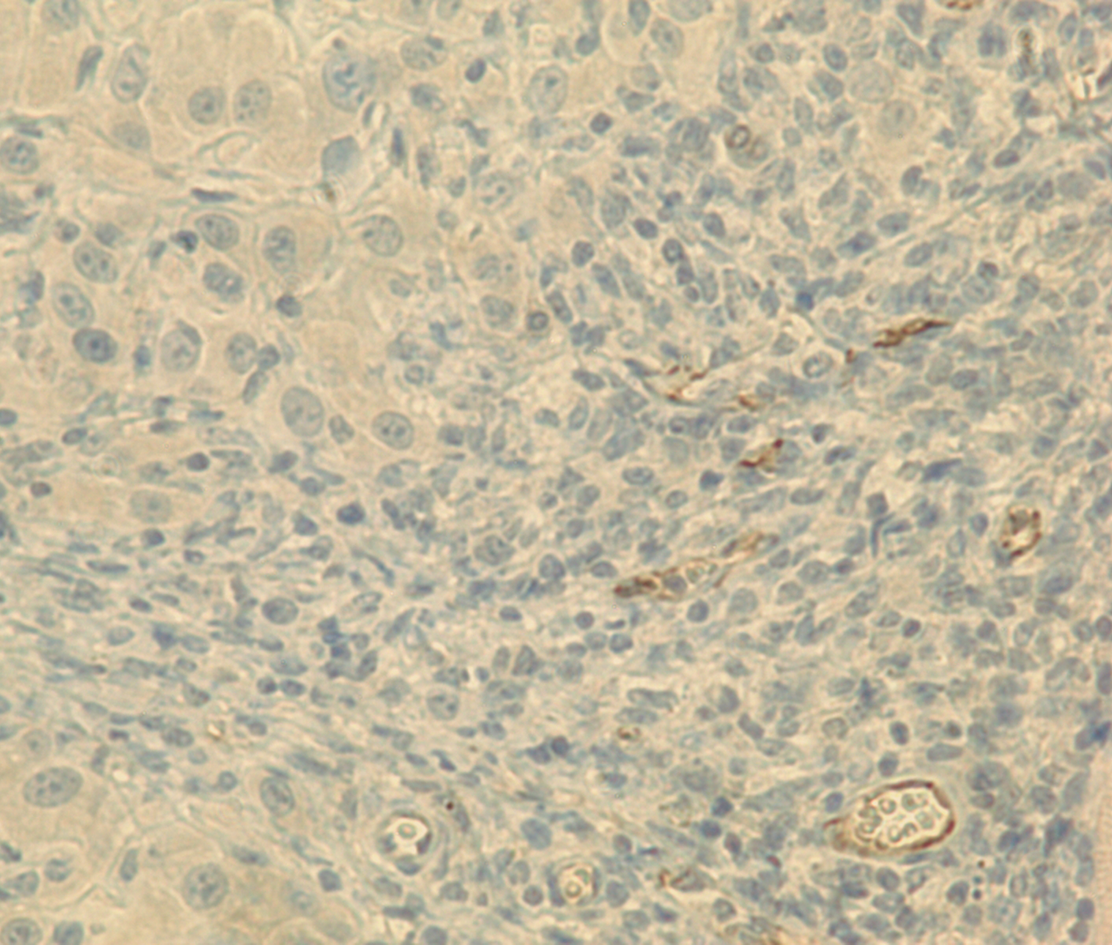

Supplement: Figure S1 — Tumour Vasculature Immunohistochemistry. Murine endothelial cells within U87 tumour were visualised by IHC staining specific for CD31. Sections were incubated with anti-PCAM-1 antibody, followed by secondary biotinylated polyclonal Rabbit anti-goat immunoglobulin, and HRP-labelled Polymer Anti-Rabbit and visualised with DAB counterstained with Harris' Hematoxylin. Endothelial cells stain brown. (TIF) [file pone.0030940.s004.tif]
